# Cross-cultural adaptation of the Karnofsky Performance Status instrument to Brazilian Portuguese

**DOI:** 10.1590/0100-6991e-20243771-en

**Published:** 2024-11-13

**Authors:** PATRÍCIA CRISTINA DOS SANTOS FERREIRA, MIRIAN NUNES MOREIRA, ROBERTO ALVES LOURENÇO

**Affiliations:** 1 - Universidade do Estado do Rio de Janeiro, Programa de Pós-graduacão em Ciências Médicas - Rio de Janeiro - RJ - Brasil; 2 - Universidade do Estado do Rio de Janeiro, Laboratório de Pesquisa em Envelhecimento Humano - Rio de Janeiro - RJ - Brasil

**Keywords:** Survival, Mortality, Prognosis, Sobrevida, Mortalidade, Prognóstico

## Abstract

**Introduction:**

The Karnofsky Performance Status (KPS) is one of the most widely used tools for assessing the prognosis of oncology patients, providing an estimate of treatment efficiency and survival. Despite this, it is commonly used in free translations without validation. The objective of the present study was to perform the cross-cultural adaptation of the KPS instrument to Brazilian Portuguese (KPS-BR) through the stages of conceptual, semantic, operational, measurement, and functional equivalences.

**Methods:**

To assess consistency, we used Cronbach’s alpha and kappa coefficients. The Chi-square test was performed to evaluate the association between scores and the number of deaths. The relationship with survival and mortality was explored with Kaplan-Meier curves.

**Results:**

A total of 316 patients participated in the study. The internal consistency analysis resulted in a Cronbach’s alpha coefficient of 0.9265. For the inter-rater analysis, the correlation coefficient was 1, as was the kappa coefficient, indicating perfect agreement between observers. The correlation coefficient between the KPS-BR scale in the test-retest was 0.8631. We observed a 100% death rate at KPS-BR scale score 20 and a gradual decrease as the KPS-BR scale score increases up to KPS-BR 40 (p<0.0001). Estimation of survival using the Kaplan-Meier method demonstrated an association between KPS-BR scale scores and survival (p<0.0001).

**Conclusion:**

The KPS-BR scale showed reliability and validity for the prognostic assessment of cancer patients, demonstrating a correlation with survival.

## RESUMO

**Introdução:**

O Karnofsky Performance Status (KPS) é um dos instrumentos mais utilizados para avaliação do prognóstico do paciente oncológico proporcionando a estimativa de eficiência do tratamento e sobrevida. Apesar disso, é comumente utilizado em traduções livres e sem validação. O objetivo do presente estudo foi realizar a adaptação transcultural do instrumento KPS para o português do Brasil (KPS-BR) em suas etapas de equivalências de conceito, semântica, operacional, mensuração e funcional.

**Métodos:**

Para avaliação da consistência utilizamos os coeficientes alfa de Cronbach e kappa. Foi realizado o teste Qui-quadrado para avaliar a associação das pontuações e o número de óbitos. A relação com o tempo de sobrevida e a mortalidade foi explorada com curvas de Kaplan-Meier.

**Resultados:**

Um total de 316 pacientes participaram do estudo. A análise de consistência interna resultou em coeficiente de confiabilidade alfa de Cronbach de 0,9265. Para análise inter-aferidor, o coeficiente de correlação foi de 1, assim como o coeficiente kappa, indicando uma concordância perfeita entre os observadores. O coeficiente de correlação entre a escala KPS-BR no teste-reteste foi de 0,8631. Observamos uma taxa de 100% de óbitos na pontuação 20 da escala KPS-BR e uma diminuição gradual à medida que a pontuação da escala KPS-BR aumenta até KPS-BR 40 (p<0,0001). A estimativa da sobrevida pelo método Kaplan-Meier mostrou uma associação entre as pontuações da escala KPS-BR e a sobrevida (p<0,0001).

**Conclusão:**

A escala KPS-BR apresentou confiabilidade e validade para a avaliação prognóstica de pacientes com câncer, mostrando associação com a sobrevida.

## INTRODUCTION

With the increase in life expectancy, the growth of chronic diseases, and the alarming estimate for the 2024-2025 biennium of 704,000 new cases of cancer per year in our country released by the National Cancer Institute (INCA)^1^, it is essential for the scientific community to discuss topics such as comprehensive evaluation of patients with oncological diseases, palliative care, and end-of-life quality of life.

The Karnofsky Performance Status (KPS) outcome measurement instrument, commonly described in our midst as a scale, is a widely used tool in the assessment of the functional status of cancer patients and is essential to guide clinical and prognostic decisions. It is an outcome prediction instrument, consisting of 11 items that vary according to the patient’s functional status, ranging from 100 (perfect health) to 0 (death). KPS was initially developed by Karnofsky et al. to assess survival after chemotherapy treatment in cancer patients^2^.

KPS was the first instrument for the evaluation of cancer patients and is considered a complement to the clinical examination. It is a scale that, in its initial version, evaluated the effects of chemotherapy on the patient’s functional level, accessing three dimensions of health: physical activity, work, and self-care. In its original description, it addressed the patient’s ability to carry out usual work activity, functionality, and dependence on constant medical care to continue living. This scale can be applied by any medical professional or multidisciplinary team. It is one of the most widely used instruments to assess the prognosis of cancer patients, providing an estimate of treatment efficiency and survival. It can be used in conjunction with other instruments to assess functional performance and survival. 

The functional decline associated with the burden of symptoms implies an increase in dependence on activities of daily living, with deterioration in quality of life and decreased survival. The assessment of functional status in cancer patients under palliative care allows the prognosis to be assessed, additional therapies to be recommended, futile and aggressive medical care to be avoided, referral to specialized care, and assessment of results of the interventions offered^3^. Although little valued by modern medicine, prognosis remains an essential parameter for clinical and surgical planning of patients. An inaccurate prognostic assessment can have disastrous consequences for patients with advanced cancer, almost as serious as an error in diagnosis or treatment^4^.

The cross-cultural adaptation of health assessment instruments is a fundamental process to ensure the validity and reliability of the measures, endorsing their applicability and accuracy in different cultural and linguistic contexts. According to the guidelines proposed by Beaton et al.^5^ and Wild et al.^6^, the cross-cultural adaptation process involves different steps, including initial translation, synthesis of translations, back-translation, review by a committee of experts, and pre-testing of the instrument in a representative sample of the target population. Within the initial translation process, it is important to consider that the Brazilian Portuguese (PT-BR) has characteristics that distance it from the European Portuguese. Therefore, special attention is needed in translation, since some scholars interpret it as a particular language^7^.

The KPS was introduced in 1948 and over the years has undergone several adjustments that carried a series of methodological problems. Inadequate adaptations of health scales - including translations with incorrect decoding of the original meaning of words, exclusion and modification of items - usually compromise the validity of these instruments^8^.

Currently, the KPS in use in most Brazilian health institutions has incorporated such methodological problems, such as customizations and free and informal translations. It is extremely important to alert the scientific community to the need of standards for KPS cross-cultural adaptation and use, avoiding the possibility of failures in patients’ prognostic evaluations. Knowledge of the KPS scale is especially necessary for surgical teams, although they are commonly involved in the early stages of oncological disease, in localized or locally advanced tumors. The surgical team may be prompted to make shared decisions with clinicians, patients, and families for care in the later stages of the disease. 

For the reasons mentioned above, the objective of the present study was to perform the cross-cultural adaptation of the KPS instrument to PT-BR, in its stages of concept, semantic, operational, measurement, and functional equivalences.

## METHODS

### Study design

This is a longitudinal study with a quantitative and descriptive approach. The cross-cultural adaptation model was based on the proposal of Herdman et al.^9^ ^,^ ^10^, with the subsequent operationalization of the process as described by Reichenheim and Moraes8. The model has the following steps: conceptual equivalence, item equivalence, semantic equivalence, operational equivalence, measurement equivalence with psychometric studies, and functional equivalence.

### The risk assessment tool

The translation and back-translation of the KPS scale represent the first four stages: conceptual equivalence, item equivalence, semantic equivalence, and operational equivalence. In the present study, two translations were performed independently. Subsequently, these versions were back-translated into the original by other independent translators. The translations from English to Portuguese were carried out by English-proficient Brazilians (standard norms of the cultured language). The back-translations were done by an American and a British, both of whom were fluent in Portuguese. The last stage was carried out with a focus group composed of specialists (a surgeon, an oncologist, a palliative care worker, a social worker, a physiotherapist, and a geriatrician). We do not consider it appropriate to perform this step with the target population, since the scale addresses a sensitive issue of prognostic assessment and patient life span. The objective of the focus group was the preparation of the pre-test version. Based on the considerations of the focus group, it was possible to make the final semantic adjustments of the synthesis version that was applied in the research (KPS-BR; Table 1). 

**Table 1:** Cross-cultural adaptation of the Karnofsky Performance Scale for Brazilian Portuguese. KPS-BR - synthesis version. 2024.

100	Normal, sem queixas, sem evidências de doença.
90	Capaz de fazer atividades habituais; mínimos sinais sintomas da doença.
80	Capaz de fazer atividades habituais com esforço; alguns sinais sintomas da doença.
70	Capaz de cuidar de si mesmo. Incapaz de fazer atividades habituais ou trabalho ativo.
60	Necessita de assistência ocasional, mas é capaz de cuidar de suas necessidades.
50	Necessita de assistência considerável e cuidados médicos frequentes.
40	Incapaz; precisa de cuidados especiais e assistência.
30	Gravemente incapacitado, cuidados institucionais, hospitalares ou equivalentes são indicados, embora a morte não seja iminente.
20	Gravemente doente, cuidados institucionais, hospitalares ou equivalentes são indicados, necessidade de tratamento com suporte ativo.
10	Morrendo, processo de morte progredindo rapidamente.
0	Morto

All patients had the ability to understand and make decisions as to sign the informed consent form (ICF), and so they did after approval by the Ethics in Research Committees (CEP) of the institutions involved, CEP HUPE UERJ (opinion number 4,770,226) and CEP INCA (opinion number 4,689,203). The sample consisted of adult individuals of both sexes with oncological pathologies and followed up at the oncology and palliative care services of the Pedro Ernesto University Hospital and at Unit IV of the National Cancer Institute, both in the city of Rio de Janeiro, state of Rio de Janeiro, Brazil. Data collection and analysis took place between 2022 and 2023. The instrument for data collection included demographic data, functional assessment using the Katz^11^ and Lawton^12^ scales for the assessment of basic activities of daily living and instrumental activities of daily living, respectively, the Edmonton Symptom Assessment System^13^ (ESAS), and our synthesis version (KPS-BR, Table 1).

To minimize the subjectivity of the high KPS-BR scores, which consider the symptoms 100 (no complaints/no evidence of disease), 90 (minimal symptoms), and 80 (some symptoms), the focus group defined an assessment of the number of symptoms through the ESAS. The researchers were told that zero symptoms would correspond to “no complaints/no evidence of disease,” one symptom would correspond to “minimal symptoms,” and two or more symptoms would correspond to “some symptoms.” This was only a filling guideline, not incorporated to the KPS-BR synthesis version.

To estimate inter-observer reliability, two observers (PF and MM) evaluated the individuals at the same time. For test-retest reliability, the same observer applied the scale in a time interval of approximately 48 hours, as suggested by the experts focus group. This time interval was considered adequate, anticipating that the patient would remain functionally stable.

### Statistics

The statistical evaluation included the equivalence of measurement with psychometric studies and functional equivalence. We performed three different types of reliability analysis of the KPS scale, namely, internal, inter-observer, and test-retest consistency. We used the Cronbach’s alpha coefficient to evaluate internal consistency. We applied the kappa coefficient to estimate the inter-observer and test-retest reliabilities.

To provide a comprehensive understanding of the distribution of KPS-BR scores and their association with survival and deaths, both in the general study population and in the subgroups of interest, we conducted several analytical steps. We generated summary statistics to describe the distribution of KPS-BR scores in relation to the two study variables: survival time and number of deaths. These statistics include median and interquartile range for survival time and frequency distribution for deaths. We performed the chi-square test to assess the association between each of the scores and the number of deaths. In addition, we used the Z test for proportions to verify whether there was a significant difference between the proportion of deaths and survivors in each of the KPS statuses. We assessed the relationship between the KPS-BR score and survival time and mortality using Kaplan-Meier survival curves. We analyzed survival data to ensure that the proportional hazards assumption was valid, especially considering the relationship to survival time. We investigated the existence of significant differences in the adjusted survival distributions using the log-rank test. In the presence of differences, we performed a post-hoc pair comparison test to determine which curves differed. We used the RStudio package version 4.2.214 for data analysis, and the significance level was set at 5%.

## RESULTS

### Descriptive analytics

Of the total of 316 patients, 170 were women (54%), with a median age of 64 years (range 23-93). Median schooling time was eight years (range 0-24). Most patients were white (45%) and brown (38%), and the remaining were 17% black and less than 1% Asian. In addition, the majority were married (34%) or single (35%), with 14% divorced, 13% widowed, and 4% in civil union. 

**Figure 1:**
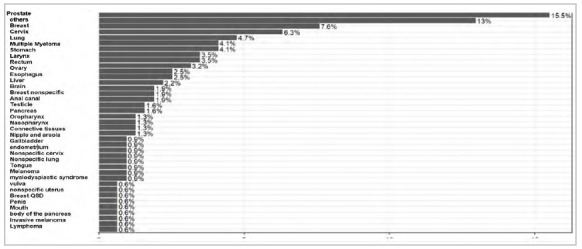
Cross-cultural adaptation of the KPS scale for the Brazilian Portuguese. Distribution of neoplasm types classified by ICD-10 (International Classification of Diseases).

Figure 1 shows prostate cancer (15.5%) as the most frequent, followed by breast cancer (7.6%). 

**Figure 2:**
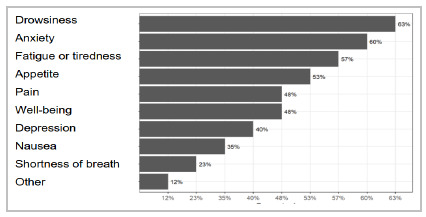
Cross-cultural adaptation of the KPS scale for Brazilian Portuguese. Distribution of symptoms. Edmonton Symptom Assessment System.

Figure 2 shows the distribution of the types of symptoms in the group of patients studied. Most patients experienced drowsiness, followed by anxiety, fatigue or tiredness, and loss of appetite.

**Figure 3:**
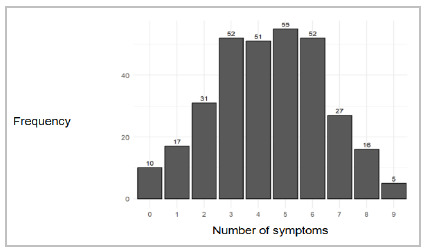
Cross-cultural adaptation of the KPS scale for Brazilian Portuguese. Distribution of the number of symptoms per patient..

Figure 3 shows the distribution of the number of symptoms per patient. The median was four symptoms, ranging from zero to nine symptoms.

### Inferential Analysis

For the internal consistency analysis, we calculated the Cronbach’s alpha reliability coefficient, which resulted in 0.9265. For the inter-observer analysis, the correlation coefficient was 1, as well as the kappa coefficient, indicating a perfect agreement between the observers. Table 2 presents summary measures of the KPS-BR scale in the test-retest analysis. 

**Table 2:** Cross-cultural adaptation of the KPS scale for Brazilian Portuguese. Brief description of the test-retest KPS-BR variables.

Variable	n	Average	Standard deviation	Min	Max
KPS-BR test KPS-BR retest	35	44.85714	15.60004	20	80
	35	41.42857	14.97898	10	80

We observed that the summary measures of the KPS-BR scale in the test-retest are similar, being on average lower in the retest. The correlation coefficient between the KPS-BR scale in the test-retest was 0.8631, i.e., there is a strong positive association between the values observed in the test-retest. The degree of kappa agreement was verified between the evaluations in the test-retest (Table 3). We observed a moderate agreement of 0.55, and the p-value indicates that the answers were not given at random, that is, we can consider the data reliable.

**Table 3:** Cross-cultural adaptation of the KPS scale for Brazilian Portuguese. Agreement between evaluations (test-retest KPS-BR).

Agreement	Expected agreement	Kappa	Standard Error	Z	p-value
65.71%	22.53%	0.5574	0.0808	6.89	0.0000

Table 4 shows the distribution of deaths and survival time, considering each of the categories of the KPS-BR scale. The median survival time for the overall sample was 68 days, with an inter-quartile range (IQR) of 13-165 days. Death occurred in 51% of patients, indicating a relatively balanced distribution between survivors and deaths in the sample.

**Table 4:** Cross-cultural adaptation of the KPS scale for Brazilian Portuguese. Association of survival and number of deaths according to the KPS-BR score.

		Survival (days)		Death n(%)		p-value (a)
KPS	n(%)	Median	(IQR)	No	Yes	
Total	316(100)	68	(13,165)	162 (51)	154 (49)	
20	11 (3)	4	(3,21)	0 (0)	11 (100)	****
30	59 (19)	12	(7,150)	14 (24)	45 (76)	****
		Survival (days)		Death n(%)		p-value (a)
KPS	n(%)	Median	(IQR)	No	Yes	
Total	316(100)	68	(13,165)	162 (51)	154 (49)	
40	69 (22)	16	(7,45)	13 (19)	56 (81)	****
50	47 (15)	43	(18,163)	17 (36)	30 (64)	0,06
60	22 (7)	115	(51,173)	15 (68)	7 (32)	0,09
70	43 (14)	110	(72,158)	38 (88)	5 (12)	****
80	42 (13)	133	(87,170)	42 (100)	0 (0)	****
90	13 (4)	170	(159,180)	13 (100)	0 (0)	****
100	10 (3)	149	(135,175)	10 (100)	0 (0)	***

a Z test for comparison of proportions; *** p-value <0.001; **** p-value <0.0001.

As can be seen in Table 5, there is an association between lower KPS-BR scores and death. We observed a 100% rate of deaths at KPS-BR score 20 and a gradual decrease as the KPS-BR score increased to 40 (p<0.0001). In addition, based on the proportion test (p-value column), we observed that only the intermediate KPS-BR scores (50 and 60) had no association with death events. In the KPS-BR scores from 70 to 90 and 100, there were associations between the scale score and the event of death (p<0.0001 and p<0.001, respectively).

**Table 5:** Cross-cultural adaptation of the KPS scale for Brazilian Portuguese. Post-hoc test of multiple comparisons considering the KPS-BR scale.

	20	30	40	50	60	70	80	90
30	0,00925	-	-	-	-	-	-	-
40	0,01978	0,63463	-	-	-	-	-	-
50	****	0,08007	0,00925	-	-	-	-	-
60	****	0,00088	****	0,02039	-	-	-	-
70	****	****	****	****	0,11274	-	-	-
80	****	****	****	****	0,00045	0,04012	-	-
90	****	****	****	0,00067	0,04012	0,26504	0,30843	-
100	****	0,00061	****	0,00298	0,07839	0,30843	0,87940	0,26527

**** p-value <0.0001.

**Figure 4:**
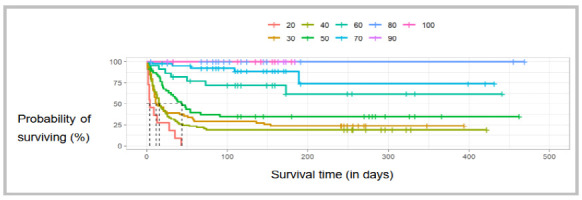
Cross-cultural adaptation of the KPS scale for Brazilian Portuguese. Estimated survival according to the Kaplan-Meier curve.

Figure 4 shows the estimated survival by the Kaplan-Meier curve according to the KPS-BR scale. There was an association between KPS-BR scores and survival (p<0.0001). Next, we applied the multiple comparison test with Bonferroni correction to compensate for multiple comparisons. Therefore, when dividing the threshold p-value of 0.05 by 8, the significance limit was set at p<0.00625. Thus, as shown in Table 5, the KPS-BR scale displayed a statistically significant difference in the survival distributions: between item 20 of the scale and items 50, 60, 70, 80, 90, and 100; between item 30 and items 60, 70, 80, 90, and 100; between item 40 and items 60, 70, 80, 90, and 100; between item 50 and items 70, 80, 90, and 100; and between item 60 and item 80 of the scale.

## DISCUSSION

The objective of the present study was to cross-culturally adapt the KPS scale to Brazilian Portuguese. From two translations and two back-translations of the original version, we reached a synthesis version that, when applied to a representative sample of cancer patients, showed good test-retest and inter-observer stability and internal consistency. In the survival evaluation, we observed a statistically significant association between the KPS-BR score and deaths.

The Brazilian version of the KPS, developed in this study, maintained the fundamental characteristics of the original version, which allows in a simple, fast, and low-cost way an estimate of the survival of cancer patients. For this reason, we conclude that the KPS-BR scale will objectively assist decision-making and avoid subjective inferences about the prognosis of cancer patients. 

Previous literature strongly suggested that approaches that carry less subjectivity could be a valuable aid to health professionals, as well as be associated with benefits to patients’ quality of life^15^ ^,^ ^16^. In a systematic review, Grare et al.^15^ suggested that in the United States, oncologists who rely on their subjective judgments to predict patient survival are often inaccurate and overly optimistic, which can result in excessively aggressive cancer treatments, including clinical and surgical approaches. For Maltoni et al.^17^, factors such as functionality, symptomatology, quality of life, and certain laboratory tests are more predictive of death than those related to tumor characteristics. For Hui et al.^18^, it is mainly in the last weeks of life that cancer patients, their caregivers, and health professionals are faced with complex and difficult decisions, where the patient’s life expectancy can be an even more important factor in the care planning process.

An important aspect of the translation of the instrument under evaluation in the present study was that, following previous suggestions in the literature^19^ ^,^ ^20^, the KPS-BR maintained a language consistent with the clinical practice of the national health communities where it is used. The KPS-BR corrected and updated the terms of the items in the instrument, bringing better understanding to health professionals. An example of the possible divergences present in the free translations in use in Brazil was the doubt about the translation of the word “moribund”, which had two possible suggested translations: “moribundo” and “morrendo”. After evaluation with Portuguese language specialists, we concluded that the term “morrendo” has greater semantic consistency and is of evident coherence for what is intended to be achieved in expressiveness. Moreover, although in Portuguese “moribundo” and “morrendo” are similar in meaning, they do not convey the same idea, the former being more strikingly scientific than the latter. We also updated and expanded the description of scores 30 and 20 regarding the need for hospital care. Given that nowadays much care can be at home or in hospice institutions, we have replaced hospital care with institutional, hospital, or equivalent care. 

We found an association between low KPS-BR scores and deaths, and high scores with few deaths. Only the scores 50 and 60 displayed no direct association between the lower score and the higher number of death events. In agreement with the literature, our data showed the association between KPS-BR scores and survival. Huang et al.^21^ showed that the KPS scale, when used by trained professionals, is a reliable measure and an independent predictor of survival. Hauser et al. presented evidence that KPS is also a predictor of oncological outcomes, in addition to predicting survival^22^. Katano et al. suggested that patients with KPS scores greater than 70 exhibited a significantly favorable survival rate^23^. Natesan et al., in a cohort study with 636 patients, showed that higher KPS scores were associated with increased overall survival and lower scores were associated with a high 30-day mortality rate^24^.

Several authors have studied patients with advanced oncological disease and have considered KPS a prognostic indicator among patients with breast cancer, and as a marker of key survival estimation among patients with brain metastases from breast cancer^25^ ^-^ ^27^. A recent study by Freeman et al. confirms that the prognostic significance of KPS extends to patients with brain metastases from breast cancer, a KPS ≤ 60 being significantly associated with shorter overall survival^28^. Regarding colon cancer with brain metastasis, several authors have also concluded that KPS was an independent prognostic factor, and patients with KPS ≥ 70 could have an additional survival benefit^29^ ^-^ ^31^. Studies in patients diagnosed with advanced prostate cancer with brain metastasis have also proven that KPS scores > 70% have a more favorable prognosis^32^ ^,^ ^33^.

Notably, we observed that the KPS-BR presented a strong internal consistency, higher than recommended, suggesting that some items may be strongly correlated, possibly redundant. Thus, future studies may address this issue to test models that group one or more scale scores. 

The present study has several strengths. This is the first Brazilian study with more than 300 patients that validated the Brazilian version of the KPS from a linguistic point of view and assessed the association of the scale with survival. We emphasize that the sample was chosen from two institutions, one academic and the other a national reference in oncology and palliative care. This ensured that the data collection procedures could be carried out properly, strictly complying with the protocols previously defined in our study project. These protocols were intensively discussed with specialists in the areas of palliative care and clinical and surgical oncology, and the field researchers were trained until they reached a high degree of agreement in their evaluations, judged from the pilot study. 

One of the positive aspects of this study was that all participants were submitted to a functional assessment, which was the basis from which they were classified within a scale score. This procedure is fundamental and, in our view, should be incorporated into clinical and surgical oncological practice, preventing patient classification from depending exclusively on the subjective perception that health professionals have about the functional capacities of their patients, as Péus et al.^34^ also pointed out.

Another feature of the study was to perform, in addition to the functional assessment, a symptom scale for all patients before filling out the KPS-BR. The information on the number of symptoms helped in the completion of scores 100 to 80. The original description of the instrument carries excessive subjectivity. To mitigate this, we used the number of symptoms to guide the completion, without the objective of incorporating it into the instrument. 

Future studies should be carried out with the aim of understanding the importance of including these assessments of symptoms and functionality in the KPS-BR through guidelines/algorithms for filling out the instrument, as already suggested in the literature^34^. 

A limitation of our work was that we did not analyze changes in functionality as an outcome. However, as in the previously mentioned studies^22^ ^,^ ^23^, we found a strong direct association between the level of severity/functionality of the scale scores and death, suggesting that this characteristic is a quality inherent to the scale, which was adequately transposed to KPS-BR during the cross-cultural adaptation phases.

## CONCLUSION

The KPS-BR scale, developed after the cross-cultural adaptation of the original KPS scale from the English to Brazilian Portuguese, showed reliability and validity for the prognostic evaluation of cancer patients, displaying association with survival. The KPS-BR scale enables the introduction of a valid instrument for appropriate therapeutic decisions and planning in clinical, surgical, oncological, and palliative care.

## References

[1] INCA Estimativa 2023 de Incidência de Câncer no Brasil.

[2] Karnofsky DA, Abelmann WH, Craver LF, Burchenal JH (1948). The use of the nitrogen mustards in the palliative treatment of carcinoma. Cancer.

[3] Dzierzanowski T, Gradalski T, Kozlowski M (2020). Palliative Performance Scale cross cultural adaptation and psychometric validation for Polish hospice setting. BMC Palliat Care.

[4] Sutherland R (2019). Dying Well-Informed The Need for Better Clinical Education Surrounding Facilitating End-of-Life Conversations. Yale J Biol Med.

[5] Beaton D, Bombardier C, Guillemin F, Ferraz MB (2000). Guidelines for the Process of Cross-Cultural Adaptation of Self-Report Measures. Spine.

[6] Wild D, Grove A, Martin M, Eremenco S, McElroy S, Verjee-Lorenz A (2005). Principles of Good Practice for the Translation and Cultural Adaptation Process for Patient-Reported Outcomes (PRO) Measures Report of the ISPOR Task Force for Translation and Cultural Adaptation. Value in Health.

[7] Bagno M (2018). Norma Linguística, Hibridismo e Tradução. Traduzires.

[8] Reichenheim ME, Moraes CL (2007). Operacionalização de adaptação transcultural de instrumentos de aferição usados em epidemiologia. Rev Saúde Pública.

[9] Herdman M, Fox-Rushby J, Badia X (1997). 'Equivalence' and the translation and adaptation of health-related quality of life questionnaires. Qual Life Res.

[10] Herdman M, Fox-Rushby J, Badia X (1998). A model of equivalence in the cultural adaptation of HRQoL instruments the universalist approach. Qual Life Res.

[11] Katz S, Ford AB, Moskowitz RW, Jackson BA, Jaffe MW (1963). Studies of illness in the aged The index of ADL: a standardized measure of biological and psychosocial function. JAMA.

[12] Lawton MP, Brody EM (1969). Assessment of older people Self-maintaining and instrumental activities of daily living. The Gerontologist.

[13] Bruera E, Kuehn N, Miller MJ, Selmser P, Macmillan K (1991). The Edmonton Symptom Assessment System (ESAS) a simple method for the assessment of palliative care patients. J Palliat Care.

[14] RStudio Team (2020). RStudio: Integrated Development for R.

[15] Glare P, Virik K, Jones M, Hudson M, Eychmuller S, Simes J, Christakis N (2003). A systematic review of physicians' survival predictions in terminally ill cancer patients. BMJ.

[16] De Borja MT, Chow E, Bovett G (2004). The correlation among patients and health care professionals in assessing functional status using the Karnofsky and eastern cooperative oncology group performance status scales. Support Cancer Ther.

[17] Maltoni M (2005). Prognostic factors in advanced cancer patients evidence-based clinical recommendations-a study by the steering committee of the European Association for Palliative Care. J Clin Oncol.

[18] Hui D (2015). Prognostication of survival in patients with advanced cancer predicting the unpredictable?. Cancer Control.

[19] Abernethy AP, Shelby-James T, Fazekas BS, Woods D, Currow D (2005). The Australia-modified Karnofsky Performance Status (AKPS) scale a revised scale for contemporary palliative care clinical practice. BMC Palliative Care.

[20] Yates JW, Chalmer B, McKegney FP (1980). Evaluation of patients with advanced cancer using the Karnofsky performance status. Cancer.

[21] Huang Y, Roy N, Dhar E, Upadhyay U, Kabir MA, Uddin M (2023). Deep Learning Prediction Model for Patient Survival Outcomes in Palliative Care Using Actigraphy Data and Clinical Information. Cancers (Basel).

[22] Hauser CA, Stockler MR, Tattersall MH (2006). Prognostic factors in patients with recently diagnosed incurable cancer a systematic review. Supp Care Cancer.

[23] Katano A, Minamitani M, Tongyu G, Ohira S, Yamashita H (2024). Survival Following Palliative Radiotherapy for Head and Neck Squamous Cell Carcinoma Examining Treatment Indications in Elderly Patients. Cancer Diagn Progn.

[24] Natesan D, Carpenter DJ, Giles W, Oyekunle T, Niedzwiecki D, Reitman ZJ (2023). Clinical Factors Associated With 30-Day Mortality Among Patients Undergoing Radiation Therapy for Brain Metastases. Adv Radiat Oncol.

[25] Sperduto PW, Mesko S, Li J, Cagney D, Aizer A, Lin NU (2020). Survival in Patients With Brain Metastases Summary Report on the Updated Diagnosis-Specific Graded Prognostic Assessment and Definition of the Eligibility Quotient. J Clin Oncol.

[26] Lee SS, Ahn JH, Kim MK, Sym SJ, Gong G, Do Ahn S (2008). Brain Metastases in Breast Cancer Prognostic Factors and Management. Breast Cancer Res Treat.

[27] Dyer MA, Kelly PJ, Chen YH, Pinnell NE, Claus EB, Lee EQ (2012). Importance of Extracranial Disease Status and Tumor Subtype for Patients Undergoing Radiosurgery for Breast Cancer Brain Metastases. Int J Radiat Oncol Biol Physics.

[28] Freeman M, Ennis M, Jerzak KJ (2022). Karnofsky Performance Status (KPS) =60 Is Strongly Associated With Shorter Brain-Specific Progression-Free Survival Among Patients With Metastatic Breast Cancer With Brain Metastases. Front Oncol.

[29] Lu X, Cai Y, Xia L (2019). Treatment modalities and relative survival in patients with brain metastasis from colorectal cancer. Biosci Trends.

[30] Bonadio RC, Freitas GF, Batista DN (2021). Epidemiology and outcomes of patients with brain metastases from colorectal cancer - who are these patients. Clin Colorectal Cancer.

[31] Li W, Wang T, Zhu Y, Yu H, Ma L, Ding Y (2022). Brain metastasis from colorectal cancer Treatment, survival, and prognosis. Medicine (Baltimore).

[32] Dziggel L, Schild SE, Veninga T, Bajrovic A, Rades D (2017). Clinical Factors Asssociated with Treatment Outcomes following Whole-brain Irradiation in Patients with Prostate Cancer. In Vivo.

[33] Tobias B, Rades D (2014). Predicting Survival after Whole-Brain Irradiation for Cerebral Metastases from Prostate Cancer. Anticancer Res.

[34] Péus D, Newcomb N, Hofer Si (2013). Appraisal of the Karnofsky Performance Status and proposal of a simple algorithmic system for its evaluation. BMC Med Inform Decis Mak.

